# Editorial: Five-membered heterocycles: synthesis and applications

**DOI:** 10.3389/fchem.2024.1445671

**Published:** 2024-06-25

**Authors:** Mohammad Abrar Alam

**Affiliations:** Department of Chemistry and Physics, College of Sciences and Mathematics, Arkansas State University, Jonesboro, AR, United States

**Keywords:** heterocycle, pyrazole, imidazole, five-member ring, thiazole

**FIGURE 1:**
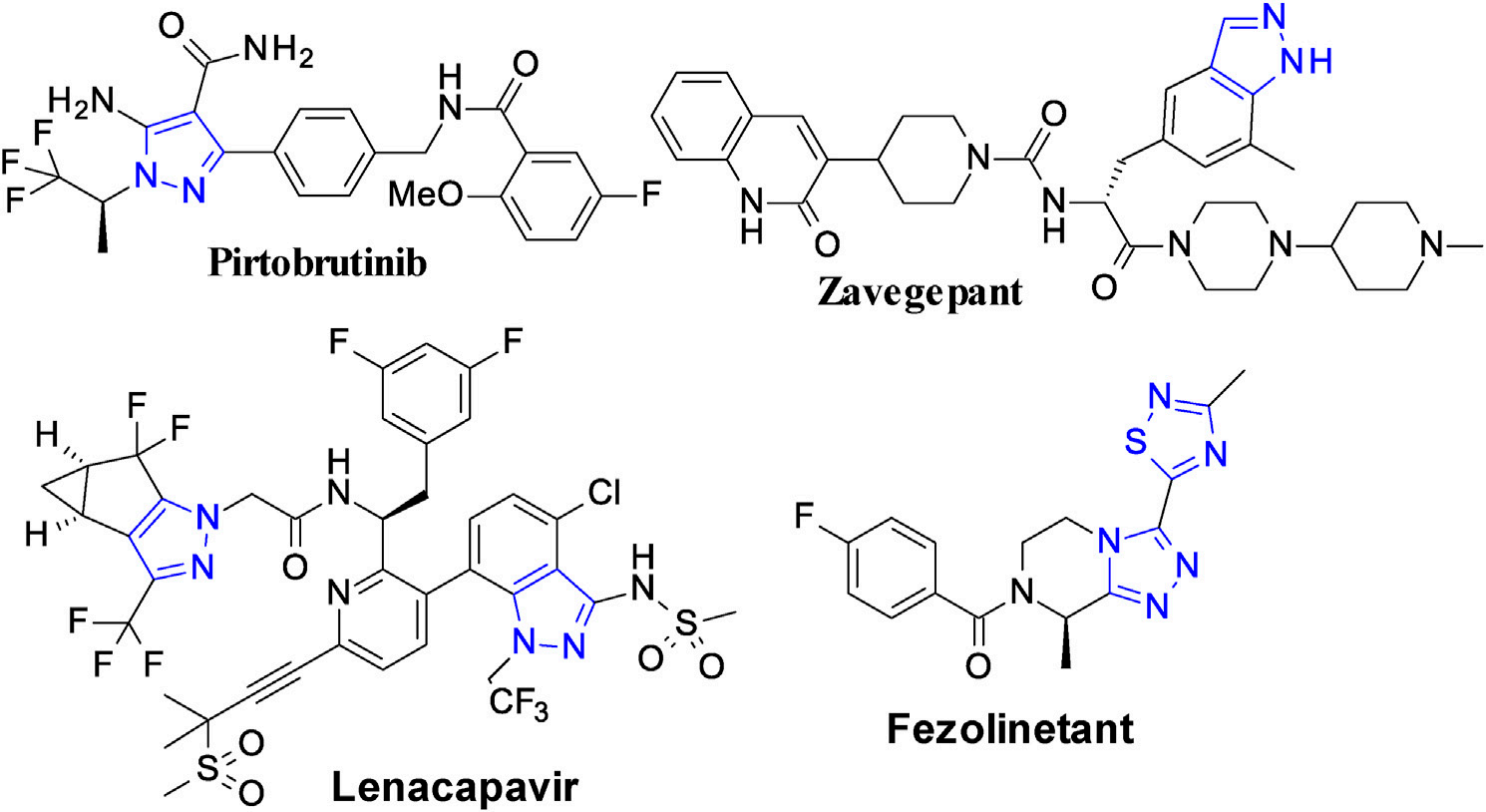
Representative examples of recently approved drugs containing five-membered azaheterocycles.

Small synthetic molecules remain prevalent as drugs both in development pipelines and on the market despite of new and innovative therapeutic approaches emerging over the years. About 90% of approved drugs are small molecules and most of these drugs contain at least one nitrogen heterocycle. These molecules easily diffuse across cell membranes to interact with the cellular organelles and proteins (Roberts et al., 2013). Several five-membered azaheterocycles are the cornerstones of various privileged scaffolds in drug discovery (Alam, 2023; Alam et al., 2023). The 1,2,3-triazole (a five-membered azaheterocycle) ring has emerged as a major pharmacophore in drug discovery. In addition to a core structure, this heterocycle is being extensively used as a “linker” to connect different pharmacophore units by using “click” chemistry (Bozorov et al., 2019). The structure of some of the most recently approved drugs containing five-membered azaheterocycle nuclei are shown in the Figure 1.

In this Research Topic, experts have contributed four articles related to the five-membered azaheterocycle small molecules.

Mangal et al. illustrated the use of ‘click chemistry’ to conjugate a bioactive natural product, zingerone with catechol, a siderophore of Gram-negative bacteria, particularly *Pseudomonas aeruginosa*. The triazole moiety acted as a linker for targeted drug delivery exploiting the bacterial iron scavenging pathway. Docking studies suggested that the hybrid compound interacted with a membrane receptor, PirA. In another research article, (Saeed et al.) reported the synthesis of theophylline-linked 1,2,4-triazole compounds under ultrasonic irradiation. Several of these chimeric compounds showed potent serine protease inhibitory activity and inhibited the growth of bacteria with minimum inhibitory concentration as low as 0.28 μg/mL.

Wang et al. have comprehensively reviewed the design, synthesis, and various biological effects of new analogs of XRP44X, an aryl pyrazole derivative developed by (Wang et al.). XRP44X is a colchicine binding site inhibitor (CBSI). The review article compiled five-membered azaheterocycles: thiadiazole, triazole, tetrazole, thiazole, oxazole, and isoxazole analogs of the pyrazole nucleus of XRP44X. The volume and lipophilicity of the *N*-aryl group determine the inhibitory property of the colchicine binding site. Sunbal et al. contributed a mini-review transition metal–catalyzed syntheses of saturated five-membered *N*-heterocycles along with other oxygen and sulfur heterocycles. Different reactions such as cycloaddition, ring-closing metathesis, and coupling are being used to synthesize these five-membered heterocycles. Cobalt, copper, iron, palladium, rhodium, and ruthenium are the most used metals for the synthesis of these bioactive compounds. Gold and iridium are also gaining attention for the synthesis of five-membered heterocycles.
